# Endoscopic ultrasound-guided enterocolostomy for small bowel obstruction following radical cystectomy with ileal neobladder reconstruction

**DOI:** 10.1055/a-2721-9342

**Published:** 2025-11-10

**Authors:** Ting Wen, Xiaoming Wang

**Affiliations:** 1 159410 Department of Gastroenterology, Panzhihua Central Hospital, Panzhihua, China

A 77-year-old male, who was previously diagnosed with bladder cancer, underwent radical cstyectomy and ileal neobladder reconstruction. The abdominal computed tomography (CT) imaging revealed incomplete small bowel obstruction ( Fig. 1 ).

**Fig. 1:**
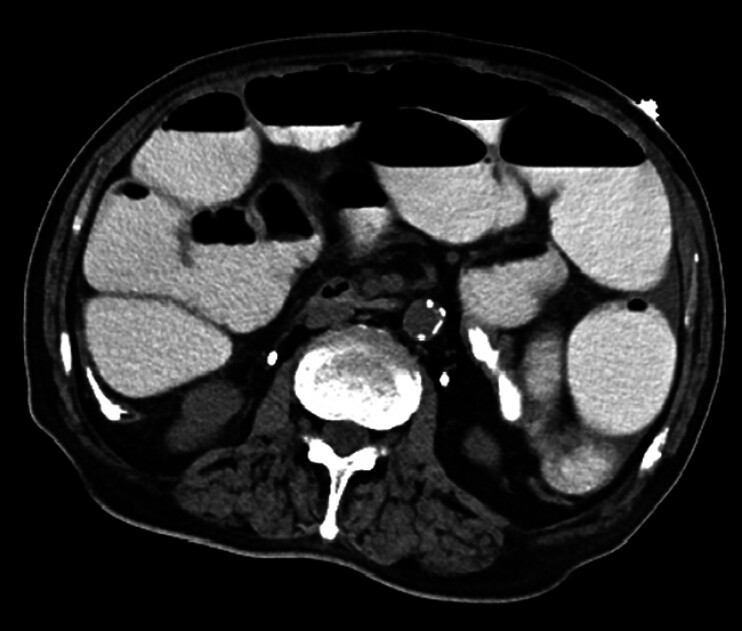
The abdominal CT imaging revealed incomplete small bowel obstruction. CT, computed tomography.

Given the patient’s cachectic state, the endoscopic ultrasound-guided enterocolostomy (EUS-EC) with a lumen-apposing metal stent (LAMS) for palliation of small-bowel obstruction was chosen ( Video 1 ). A guidewire was placed in the intestinal lumen via colonoscopy, and an ultrasound colonoscope was inserted along the guidewire. The puncture site was determined where the distance between the sigmoid colon and the dilated plane above the small intestinal obstruction was less than 1 cm. A 19-G needle was used to puncture the dilated intestinal lumen from the localization point, and the intestinal fluid was visible in the retraction, which was reconfirmed by iodinohydrol contrast as a dilated small bowel. A 15 mm × 10 mm LAMS was placed using the 150-W pure-cut mode ( Fig. 2 ).

Endoscopic ultrasound-guided enterocolostomy with LAMS for palliation of small-bowel obstruction. LAMS, lumen-apposing metal stent.Video 1

**Fig. 2:**
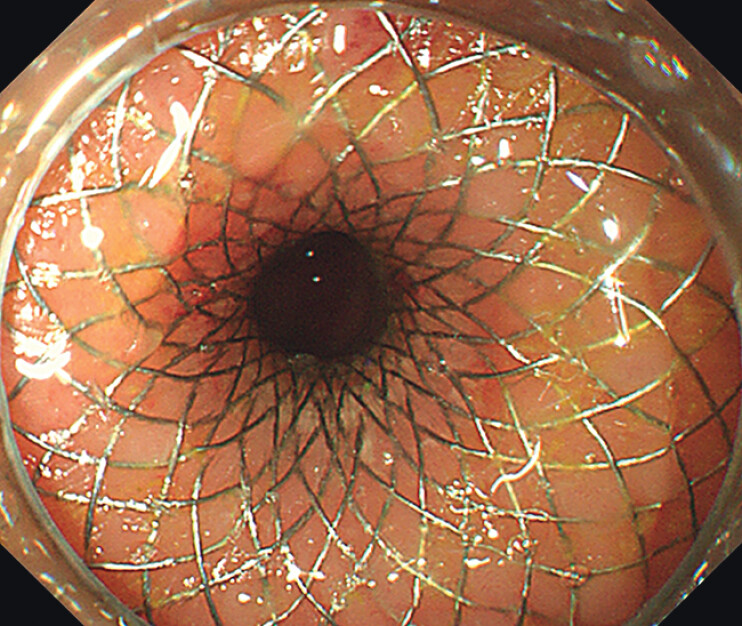
The LAMS was placed Successfully through EUS-EC. LAMS, lumen-apposing metal stent.

A follow-up abdominal CT imaging on the second postoperative day showed that the small bowel obstruction was significantly reduced compared with the previous one ( Fig. 3 ). The patient’s abdominal pain and bloating were significantly relieved. He was able to take oral nutritional supplements, and was gradually transitioning to a liquid diet.

**Fig. 3:**
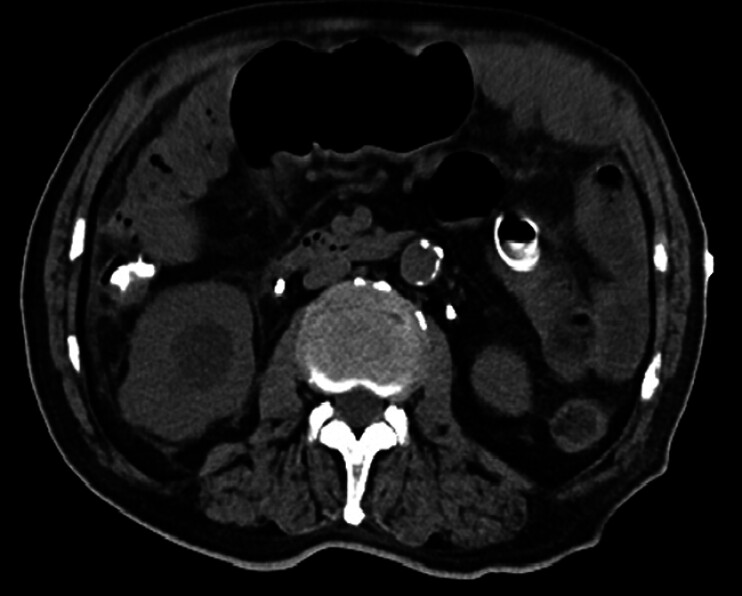
The follow-up abdominal CT imaging showed that the small bowel obstruction was significantly reduced. CT, computed tomography.

There are already relevant literature reports on common bile duct-duodenal anastomosis for obstructive distal common bile duct, gastrointestinal anastomosis for gastric outlet obstruction, and jejunojejunostomy for bypass surgery-induced stenosis 1 2 3 . This is the first instance of small bowel obstruction following radical cystectomy and ileal neobladder reconstruction treated with EUS-EC. This case confirms the feasibility of EUS-EC treatment for small bowel obstruction, but caution should be exercised when selecting the puncture site.

Endoscopy_UCTN_Code_TTT_1AS_2AG

## Notes

Endoscopy E-Videos https://eref.thieme.de/e-videos
*E-Videos* is an open access online section of the journal *Endoscopy* , reporting on interesting cases and new techniques in gastroenterological endoscopy. All papers include a high-quality video and are published with a Creative Commons CC-BY license. Endoscopy E-Videos qualify for HINARI discounts and waivers and eligibility is automatically checked during the submission process. We grant 100% waivers to articles whose corresponding authors are based in Group A countries and 50% waivers to those who are based in Group B countries as classified by Research4Life (see: https://www.research4life.org/access/eligibility/ ). This section has its own submission website at https://mc.manuscriptcentral.com/e-videos .
